# Agrin and Perlecan Mediate Tumorigenic Processes in Oral Squamous Cell Carcinoma

**DOI:** 10.1371/journal.pone.0115004

**Published:** 2014-12-15

**Authors:** Rebeca Kawahara, Daniela C. Granato, Carolina M. Carnielli, Nilva K. Cervigne, Carine E. Oliveria, César A. R. Martinez, Sami Yokoo, Felipe P. Fonseca, Marcio Lopes, Alan R. Santos-Silva, Edgard Graner, Ricardo D. Coletta, Adriana Franco Paes Leme

**Affiliations:** 1 Laboratório de Espectrometria de Massas, Laboratório Nacional de Biociências, LNBio, CNPEM, Campinas, Brazil; 2 Faculdade de Odontologia de Piracicaba, Universidade Estadual de Campinas, UNICAMP, Piracicaba, Brazil; University of Birmingham, United Kingdom

## Abstract

Oral squamous cell carcinoma is the most common type of cancer in the oral cavity, representing more than 90% of all oral cancers. The characterization of altered molecules in oral cancer is essential to understand molecular mechanisms underlying tumor progression as well as to contribute to cancer biomarker and therapeutic target discovery. Proteoglycans are key molecular effectors of cell surface and pericellular microenvironments, performing multiple functions in cancer. Two of the major basement membrane proteoglycans, agrin and perlecan, were investigated in this study regarding their role in oral cancer. Using real time quantitative PCR (qRT-PCR), we showed that agrin and perlecan are highly expressed in oral squamous cell carcinoma. Interestingly, cell lines originated from distinct sites showed different expression of agrin and perlecan. Enzymatically targeting chondroitin sulfate modification by chondroitinase, oral squamous carcinoma cell line had a reduced ability to adhere to extracellular matrix proteins and increased sensibility to cisplatin. Additionally, knockdown of agrin and perlecan promoted a decrease on cell migration and adhesion, and on resistance of cells to cisplatin. Our study showed, for the first time, a negative regulation on oral cancer-associated events by either targeting chondroitin sulfate content or agrin and perlecan levels.

## Introduction

Head and neck cancers are the sixth most common malignancy in the world, accounting for more than 500,000 new cases every year [1]. Oral squamous cell carcinoma (OSCC) is the most prevalent cancer occurring in this area [2]. Despite advancements in prevention and multimodality treatments, oral cancer is still characterized by poor prognosis and a low survival rate [3]–[5].

Long-standing as well as recent data implicate tumor extracellular matrix (ECM) as a significant contributor to tumor progression [6], [7]. However, the entire process orquestrated by interactions between cancer cells and ECM remains poorly understood. One of the major constituents of the ECM, the proteoglycans (PGs), is markedly altered during malignant transformation and tumor progression. Their role is associated with a number of tumorigenic processes, including control of cell growth and survival, induction of apoptosis, adhesion, and invasion [8]–[10]. Among the main heparan sulfate PGs (HSPG), identified in basement membrane, are agrin and perlecan, which not only were reported to be overexpressed in some cancers, such as prostate cancer [11], hepatocellular carcinoma [12] and breast cancer [13], but also had their function associated with tumorigenic events [10], [14], [15]. Though, no evidence was reported regarding their role in oral cancer.

Perlecan is a large proteoglycan (400–500 kDa) harboring five distinct structural domains, to which long chains of heparan sulfate and/or chondroitin sulfate are attached [16]. This molecule is present in all vascularized tissues with a distribution that is primarily confined to basement membranes [17], [18]. Also, other studies have also identified perlecan in the stromal spaces of various pathophysiological conditions [19]–[21].

Agrin shares a rather intriguing multimodular organization with perlecan, but more complexity to agrin can be added due to at least four sites of alternative splicing [22]. The amino acid sequence of agrin encodes a protein with a molecular size of 220 kDa, but the observed molecular weight is around 400 kDa due to the long heparan sulfate (HS) and chondroitin sulfate (CS) glycosaminoglycans (GAGs) attached to the core protein [23]. Although originally discovered in the neuromuscular junctions, agrin has been observed in numerous other tissues, and it is described as highly expressed in hepatocellular carcinomas [12], [24], [25] and cholangiocellular carcinomas [12], [24]. Nevertheless, little is known about its role at locations other than the neuromuscular junctions, and even less information is known about its role in tumor tissues.

In the present study, we focused on understanding the role of the proteoglycans agrin and perlecan in oral cancer. First, we sought to validated the overexpression of agrin and perlecan in oral cancer tissues compared to normal tissues and in cell lines with different site of origin: oral squamous carcinoma originated from human tongue (SCC-9), oral squamous carcinoma SCC-9 isolated from lymph nodes (SCC-9 LN-1) and a skin-derived squamous carcinoma (A431). Next, we showed that oral squamous carcinoma cell line had a reduced ability to adhere to extracellular matrix proteins and increased sensibility to cisplatin when treated with chondroitinase. By specific target agrin and perlecan protein levels with siRNA, we showed that OSCC cells have decreased cell adhesion and migration and increased sensibility to cisplatin treatment. Overall, our findings opened new avenues to better understand the role of agrin and perlecan, as well as their involvement in carcinogenesis, which may offer a novel approach to cancer therapy by targeting the tumor microenvironment.

## Materials and Methods

### Cell culture

SCC-9 cells (a tumor cell line originated from a human tongue squamous cell carcinoma) were obtained from the American Type Culture Collection (ATCC, Manassas, VA) and cultured in DMEM/Ham’s F12 medium (Cultilab), supplemented with 10% fetal bovine serum (FBS), antibiotics and 0.4 µg/ml hydrocortisone.

Metastatic oral squamous cell carcinoma (SCC-9) cells were isolated from lymph nodes (LN-1) originating the cell line SCC-9 LN-1 [26] and cultured as recommended for SCC-9. Human epidermoid carcinoma, A431, were grown in Roswell Park Memorial Institute (RPMI) −1640 medium supplemented with 10% FBS and antibiotics. All cell lines were maintained at 37°C in a 5% CO_2_ atmosphere.

### Analysis of mRNA Expression levels

Fresh-frozen OSCC samples (n = 16) and normal oral mucosa (n = 16) were used for perlecan and agrin mRNA levels quantification, using qRT-PCR. All patients and volunteers enrolled signed a formulary stating their awareness and consent for the study, approved by the Research Ethics Committee of Faculdade de Odontologia de Piracicaba, Universidade Estadual de Campinas, UNICAMP, Piracicaba, Brazil. Clinical pathological data, such as sex, age, anatomical site of the primary tumor, clinical stage and histopathological grade were collected from patient’s charts and showed in S1 Table. Brief, total mRNA was isolated from fresh-frozen tissue samples using mirVana miRNA Isolation Kit (Ambion), according to the manufacturer’s protocol. First-strand cDNAs were synthesized from 2µg of DNase-treated total RNA using the SuperScript II Reverse Transcription Kit (Invitrogen, Carlsbad, CA, USA). Briefly, diluted cDNA product (1∶3) was used to perform a quantitative reverse-transcription polymerase chain reaction (qRT-PCR) using SYBR Green PCR Master Mix (Applied Bios stems) in a 7900 Real-Time PCR System (Applied Biosystems, Foster, CA, USA). The following primers were used to determine mRNA expression levels: Perlecan forward 5′- AAT GCGCTGGACACATTCG-3′ and reverse 5′-ATTCACCAGGGCTCGGAAATA-3′; Agrin forward 5′TTGTCGAGTACCTCAACGCT-3′ and reverse 5′-CAGGCTCAGTTCAAAGTCGT-3′. PPIA (cyclophilin A) was used as the reference gene, forward 5′ GCTTTGGGTCCAGGAATGG 3′ and reverse 5′ GTTGTCCACAGTCAGCAATGGT 3′. The PCR cycles were 95°C for 10 minutes, followed by 40 cycles of 95°C for 15 seconds and 60°C for 1 minute. Each reaction was performed in triplicate and analyzed individually. The results were calculated using 2^−ΔΔCt^ relative quantification method; relative quantification was normalized to the pooled normal oral tissues, used as reference control.

For analysis of mRNA expression levels in A431, SCC-9 LN-1 and SCC-9 cell lines, total RNA was obtained using the TRIzol reagent (Invitrogen Corporation) and 2 µg of total RNA were used for retro-transcription using a First-Strand cDNA Synthesis Kit (GE Healthcare). Real-time quantitative PCR for agrin and perlecan was performed using the SYBRH Green PCR Master Mix (Applied Biosystems), and the dissociation curves were performed to confirm the specificity of the products. The threshold cycle (CT) values of the targeted gene were normalized relative to glyceraldehyde-3-phosphate dehydrogenase gene, and relative expression ratios were calculated using the 2−ΔΔ Ct method. Two independent experiments were performed with triplicates.

### Immunohistochemistry

High density tissue microarrays were obtained from Biomax (OR601a). The presence of agrin was analyzed in 10 cancer-adjacent normal tissues and in 47 primary oral squamous cell carcinomas by immunohistochemistry using the streptavidin-biotin peroxidase complex (Dako). Protein quantification was assessed with the aid of Aperio Scanscope CS Slide Scanner and the Pixel Count V9 algorithm software (Aperio Technologies, Vista, CA; USA). By using specific input parameters, the percentage of cytoplasm positivity was calculated and classified as weak, moderate and strong, according to its staining intensity. Each category received an intensity score, 1 to weak, 2 to moderate and 3 to strong staining. The final score of each tissue sample was calculated as the sum of the percentage of each category multiplied by their respective intensity scores as the following formula: Score = (%weak×1)+(%moderate×2)+(%strong×3). Clinical pathological data, such as sex, age, anatomical site of the primary tumor, clinical stage and histopathological grade were collected from patient’s charts and showed in S2 Table.

### Chondroitinase treatment

Chondroitinase ABC from Proteus vulgaris (Sigma) was reconstituted in a 0.01% bovine serum albumin (BSA) aqueous solution to a final concentration of 6 U/ml. The treatment was performed by diluting the stock chondroitinase in serum free media to a final concentration of 0.1 U/ml for 4 h at 37°C. After treatment, SCC-9 LN-1 cell lines were submitted to adhesion and migration assays.

### In vitro cell migration and adhesion assays

SCC-9, SCC-9 LN-1 or A431 cells, transfected with control siRNA (scramble, sc-44510, Santa Cruz) or specific siRNA against agrin (sc-29652, Santa Cruz) or perlecan (sc-44010, Santa Cruz), were submitted either to cell migration or adhesion assays. Briefly, 3×10^5^ cells were plated in 6 well-plate and, after 24 h, the oligoribonucleotides were transfected with lipofectamine 2000, according to the manufacturer (Invitrogen).

For the in vitro cell migration assay, cells were harvested after 72 h, plated in the upper chambers of the transwell (HTS Transwell 96-Well Plate, Corning), and allowed to migrate towards the lower chamber containing RPMI medium supplemented with 1% FBS for 16 h. At the end of the assay, the non-migrated cells at the top chamber were removed using a cotton swab, and the cells at the bottom of the insert filter were fixed with 10% formaldehyde for 10 min, followed by PBS washing and then staining with 1% toluidine blue solution in 1% borax, for 5 min. The dye was eluted using 1% SDS, and the absorbance of stained cells was measured at 620 nm. Three independent experiments were performed in triplicates.

SCC-9, SCC-9 LN-1 or A431 siRNAs-transfected cells were also submitted to cell adhesion assay, as described by Aragão et al [27]. After 72 h, cells were seeded in a 6-well plate (2×10^5^) and incubated for 24 h. Then, were washed twice, incubated in serum-free media for 4 h, and seeded in a Matrigel (2 µg per well; BD Biosciences) coated 96-well plate, previously three times PBS-washed and blocked with 3% BSA during 2 h. The adhesion was evaluated during 1 h in serum-free media supplemented with 3% BSA. The wells were washed 3 times, and cells fixed with 10% formaldehyde. Cells were then stained with toluidine blue solution, and absorbance measured at 620 nm, as described above. Three independent experiments were performed with three replicates.

### In vitro viability assay

SCC-9, SCC-9 LN-1 or A431 siRNA-transfected cells (scramble, Agrin or Perlecan) were seeded onto 96-well plates and incubated at 37°C/5%CO_2_ for two days. MTT (12 mM tetrazolium 3-(4,5- dimethylthiazol-2)-2,5-diphenyltetrazolium bromide) was added, and cells were kept at 37°C for 4 h, in the dark. The media was removed, and 100 µl of 1 N HCl/isopropanol (1∶25) was added into each well, followed by gentle agitation at room temperature for 15 min. Finally, the absorbance was measured at 595 nm. Three independent experiments were performed in triplicates.

### Drug Sensitivity Assay (MTT)

Cisplatin [cis-diammineplatinum(II) dichloride] (Sigma-Aldrich) was dissolved in 0.150 M NaCl. Aliquots were stored at −20**°**C for up to a maximum of three months, and thawed immediately before use.

Cells (1×10^4^) were seeded in 96-well plates and allowed to adhere overnight at 37°C. Briefly, following treatments of cells with cisplatin (0; 1; 5; 7.5; 10; 25; 50; 100 µM) for 48 h, MTT reagent [3-(4,5-Dimethylthiazol-2-yl)-2,5-diphenyltetrazolium bromide] was added to each well, and incubated for 4 h at 37°C, in the dark. The media were removed, 100 µl of 1 N HCl/isopropanol (1∶25) was added in each well, and incubated for 15 min at room temperature under gentle agitation. Finally, absorbance was measured at 595 nm. Three independent experiments were performed in triplicates.

### Statistical Analysis

Statistical analysis of gene expression validation by qRT-PCR and immunohistochemistry was performed using the non-parametric Kruskal-Wallis followed by Dunn’s test and Mann-Whitney U test, respectively. For the functional assays adhesion, migration and proliferation, Student’s *t*-test or ANOVA followed by Tukey’s test was used; p-values<0.05 were set as statistically significant. For drug sensitivity assay, a nonlinear regression curve fit (one phase exponential decay) was used to analyze cisplatin dose response experiments and determine the IC50. All the statistical analysis for mRNA expression analysis and functional assays were performed in GraphPad Prism v6.01.

## Results

### Agrin and Perlecan are up-regulated in OSCC samples

**Figure 1:**
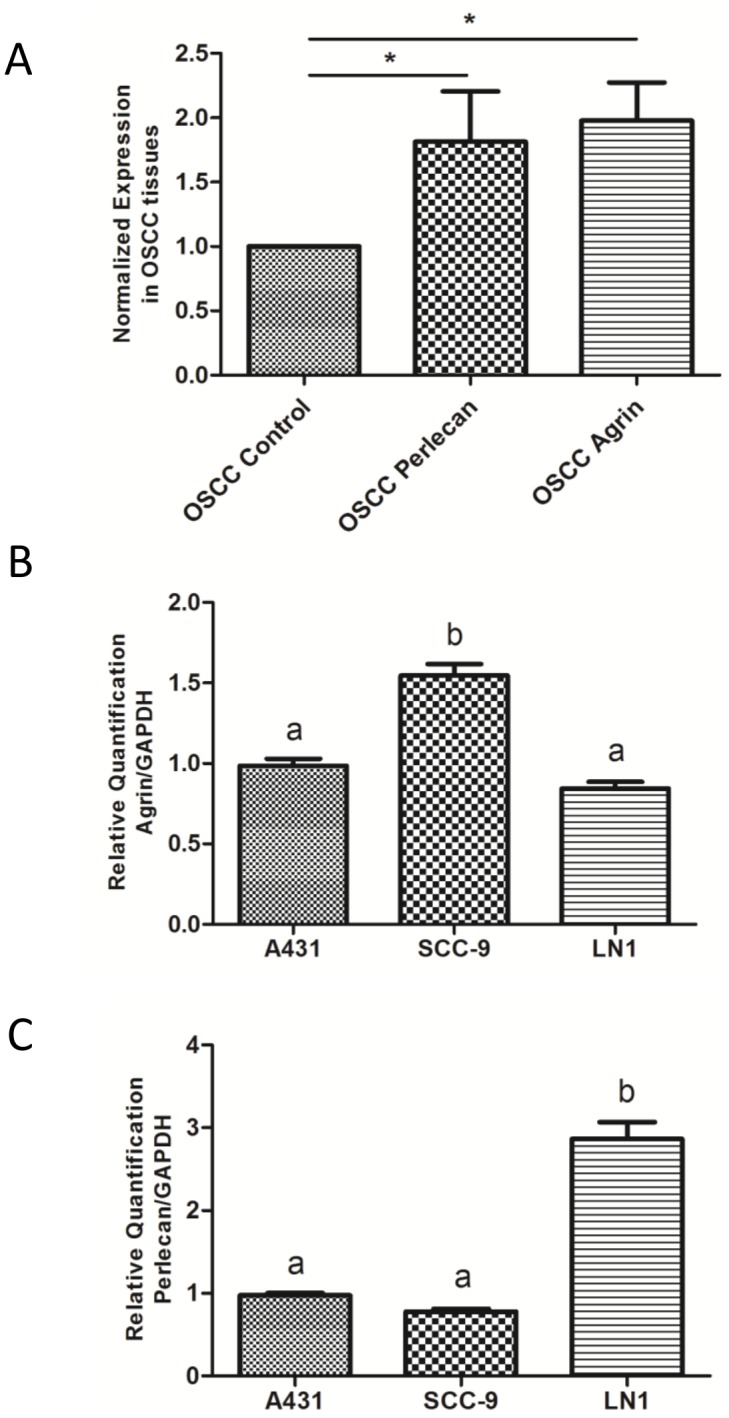
mRNA expression levels of agrin and perlecan. (A) Validation of higher expression of agrin and perlecan by qRT-PCR in OSCC tumor tissues**.** Agrin and Perlecan showed higher mRNA expression levels in human OSCC tumor tissues compared to control tissues by qRT-PCR (Kruskal-Wallis followed by Dunn’s test, n = 16, *p<0.05). (B) Agrin showed higher mRNA expression levels in SCC-9 compared to A431 and SCC-9 LN-1 cell lines. (C) Perlecan showed higher mRNA expression levels in SCC-9 LN-1 compared with A431 and SCC-9 cell lines. The data were normalized with glyceraldehyde-3-phosphate dehydrogenase gene, used as internal reference). Each bar represents means ± SD of at least two independent experiments in triplicates (one-way ANOVA followed by Tukey’s test. Different letters indicate statistically difference at p<0.05).

We started this study by evaluating the mRNA levels of agrin and perlecan, using qRT-PCR in an independent cohort of OSCC samples and normal oral mucosa. Higher expression levels of agrin and perlecan were observed in OSCC samples compared to controls (Fig. 1A, n = 16, Kruskal-Wallis followed by Dunn’s test, p<0.05, S1 Table).

We also performed immunohistochemistry analysis in commercial tissue microarrays containing 10 cancer-adjacent normal tissues and 47 primary OSCCs. Normal epithelial cells showed weak reactivity for the antibody against agrin, but a broad positivity was found in the neoplastic cells. Furthermore, there was a significantly higher expression of agrin in tumor samples, compared to controls (S1 Figure, Mann-Whitney U test, p<0.0005, S2 Table).

The mRNA levels of agrin and perlecan were evaluated in cell lines with different sites of origin: oral squamous carcinoma cell line (SCC-9) isolated from tongue, oral squamous carcinoma cell line (SCC-9) isolated from lymph nodes (SCC-9 LN-1) and a skin-derived squamous carcinoma (A431). Whereas the mRNA levels of agrin were higher in SCC-9, the mRNA levels of perlecan were higher in metastatic SCC-9 LN-1 cell lines. A431 showed an intermediary expression for both agrin and perlecan (Fig. 1B/C).

### Targeting chondroitin sulfate modification reduced the ability of SCC-9 LN-1 cells to adhere to extracellular matrix and decreased the cell resistance to cisplatin

**Figure 2:**
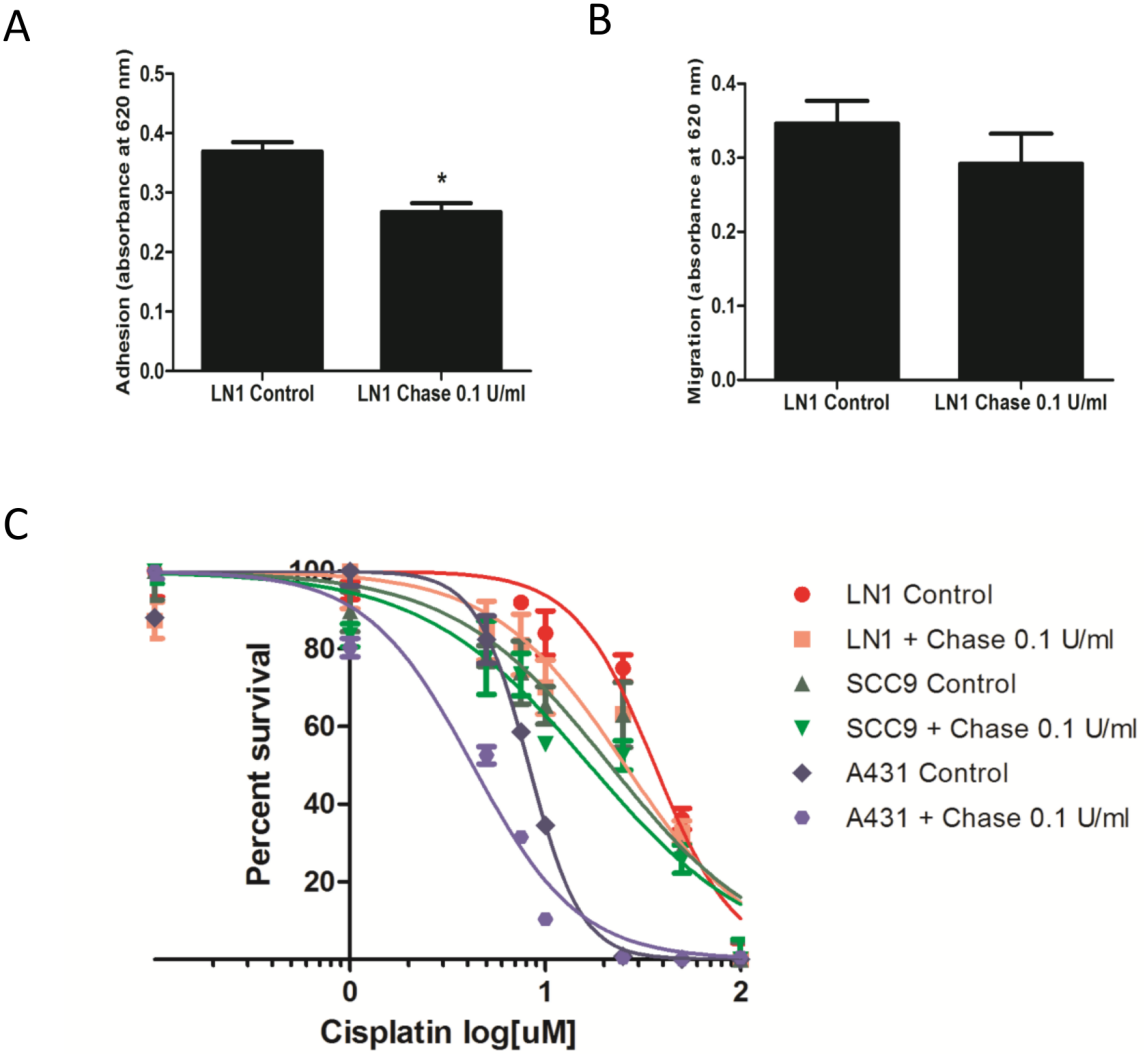
Treatment with chondroitinase ABC decreased SCC-9 LN-1 cell adhesion to extracellular matrix, but not SCC-9 LN-1 cell migration and increased sensibility of SCC-9 LN-1, SCC-9 and A431 cells to cisplatin. (A) SCC-9 LN-1 had a lower ability to adhere to extracellular matrix proteins (Matrigel) after treatment with 0.1 U/ml of chondroitinase for 4 h/37°C in serum free media (n = 3, triplicate, Student’s *t*-test, * indicates p<0.05). (B) SCC-9 LN-1 was treated with 0.1 U/ml for 4 h/37°C in serum free media were seeded in the upper chamber of 96-well transwell plates (n = 3, triplicate). RPMI media, which was supplemented with 1% FBS, was added in the lower chamber. (C) SCC-9 LN-1, SCC-9 and A431 cells treated with increasing concentrations of cisplatin (0–100 µM) for 48 h in the presence of 0.1 U/ml of chondroitinase showed increased sensibility to cisplatin, calculated by a non-linear regression of a dose-response curves (log[µM cisplatin] vs normalized response). Data are expressed as means ± SD from one independent experiment.

To further understand the general role of chondroitin sulfate post-translational modification in tumorigenic processes such as cell adhesion and migration, SCC-9 LN-1 cells were treated with 0.1 U/ml of chondroitinase for 4 h in serum free media, and evaluated for the ability of these cells to adhere to ECM proteins (Matrigel) and migrate. We showed that SCC-9 LN-1 cells treated with chondroitinase had lower ability to adhere to ECM proteins compared to the control (Fig. 2A, n = 3, Student’s *t*-test, p<0.05), whereas no significant effect was observed in the migration of SCC-9 LN-1 cells treated with chondroitinase (Fig. 2B, n = 3, Student’s *t*-test, p>0.05).

Considering previous studies demonstrated the function of heparan sulfate modifications in drug uptake [28], we also evaluated the effect of chondroitinase treatment in the cisplatin cell resistance calculated by the IC50. For that, viability of the SCC-9, SCC-9 LN-1 and A431 cells were determined in the absence (vehicle) or presence of 1, 5, 7.5, 10, 25, 50 or 100 µM of cisplatin. The same curve was constructed with the addition of 0.1 U/ml of chondroitinase. The IC50 of SCC-9 LN-1, SCC-9 and A431 was 36.31 µM, 21.5 µM and 8.24 µM, respectively. When chondroitinase was added, SCC-9 LN-1, SCC-9 and A431 cell lines exhibited a drop in the IC50 (25.62 µM for SCC-9 LN-1, 16.88 µM for SCC-9 and 4.52 µM for A431), decreasing resistance to cisplatin in about 1.5, 1.3 and 1.8 fold for SCC-9 LN-1, SCC-9 and A431 cells, respectively (Fig. 2C).

### Expression-associated phenotype of agrin and perlecan in adhesion, migration and proliferation event

**Figure 3:**
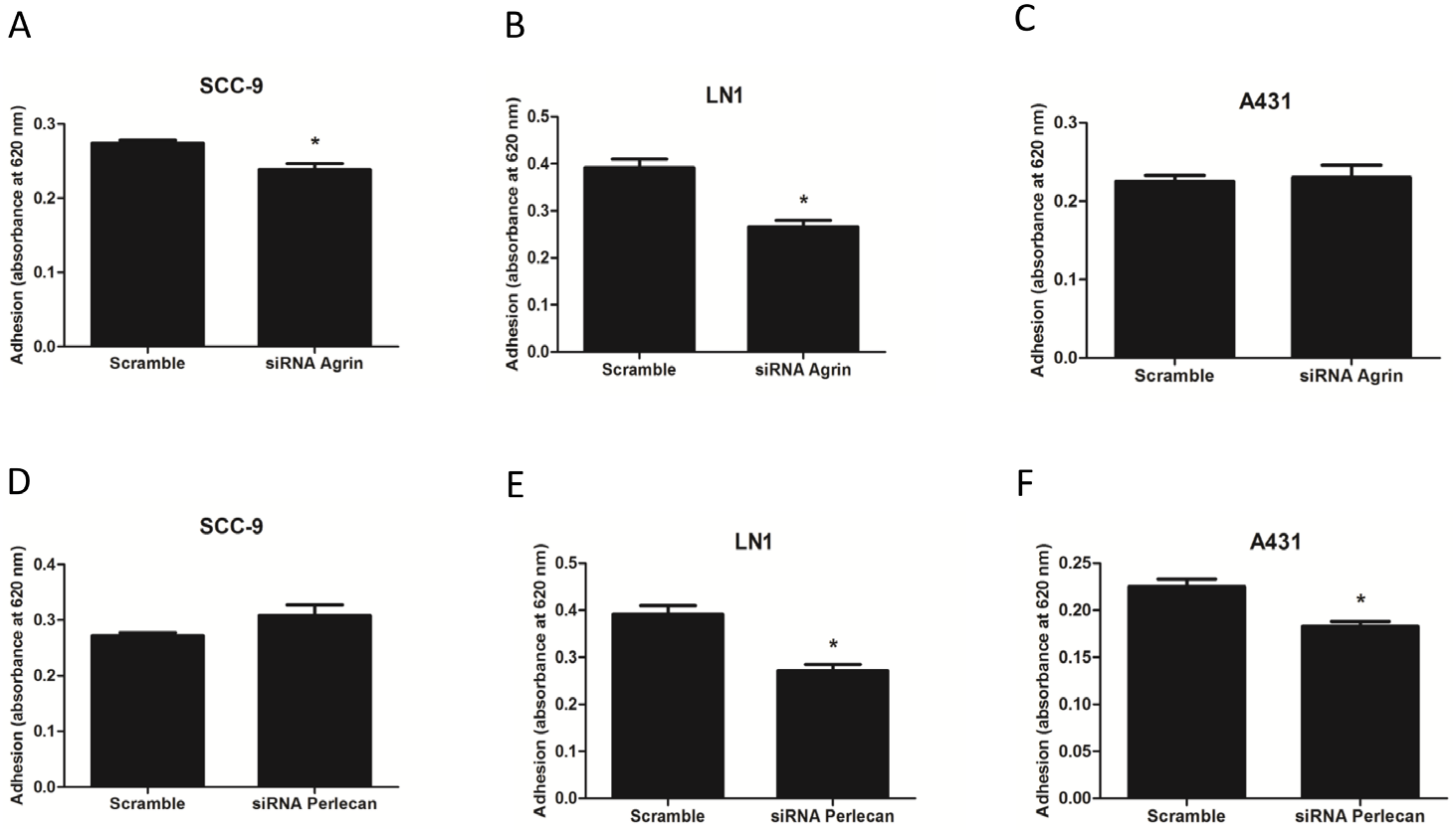
The role of agrin and perlecan in cell adhesion. Knockdown of agrin decreased adhesion to Matrigel in SCC-9 (n = 2, A) and SCC-9 LN-1 (n = 3, B), while no difference was observed in A431 (n = 3, C). When perlecan was silenced by siRNA no difference was observed in SCC-9 adhesion to Matrigel (n = 2, D) but a significant reduction was observed in SCC-9 LN-1 (n = 3, E) and A431 (n = 3, F) adhesion to Matrigel (Student’s *t*-test, * indicates p<0.05).

In order to investigate the role of agrin and perlecan in oral carcinogenesis, we first evaluated the ability of the cells to adhere to extracellular matrix proteins. First, SCC-9, SCC-9 LN-1 and A431 cells lines were scramble-siRNA, agrin-siRNA or perlecan-siRNA transfected, and knockdown of agrin and perlecan were confirmed by qRT-PCR (S2 Figure). We observed that targeted agrin knockdown decreased the adhesion of SCC-9 and SCC-9 LN-1 cells to the Matrigel (Fig. 3A and B, n = 3, Student’s *t*-test, p<0.05), but no significant effect was observed for agrin knockdown in A431 cells. The knockdown of perlecan decreased the adhesion of SCC-9 LN-1 and A431 cells (Fig. 3C and D, n = 3, Student’s *t*-test, p<0.05), but no significant effect was observed for perlecan knockdown in SCC-9 cells.

**Figure 4:**
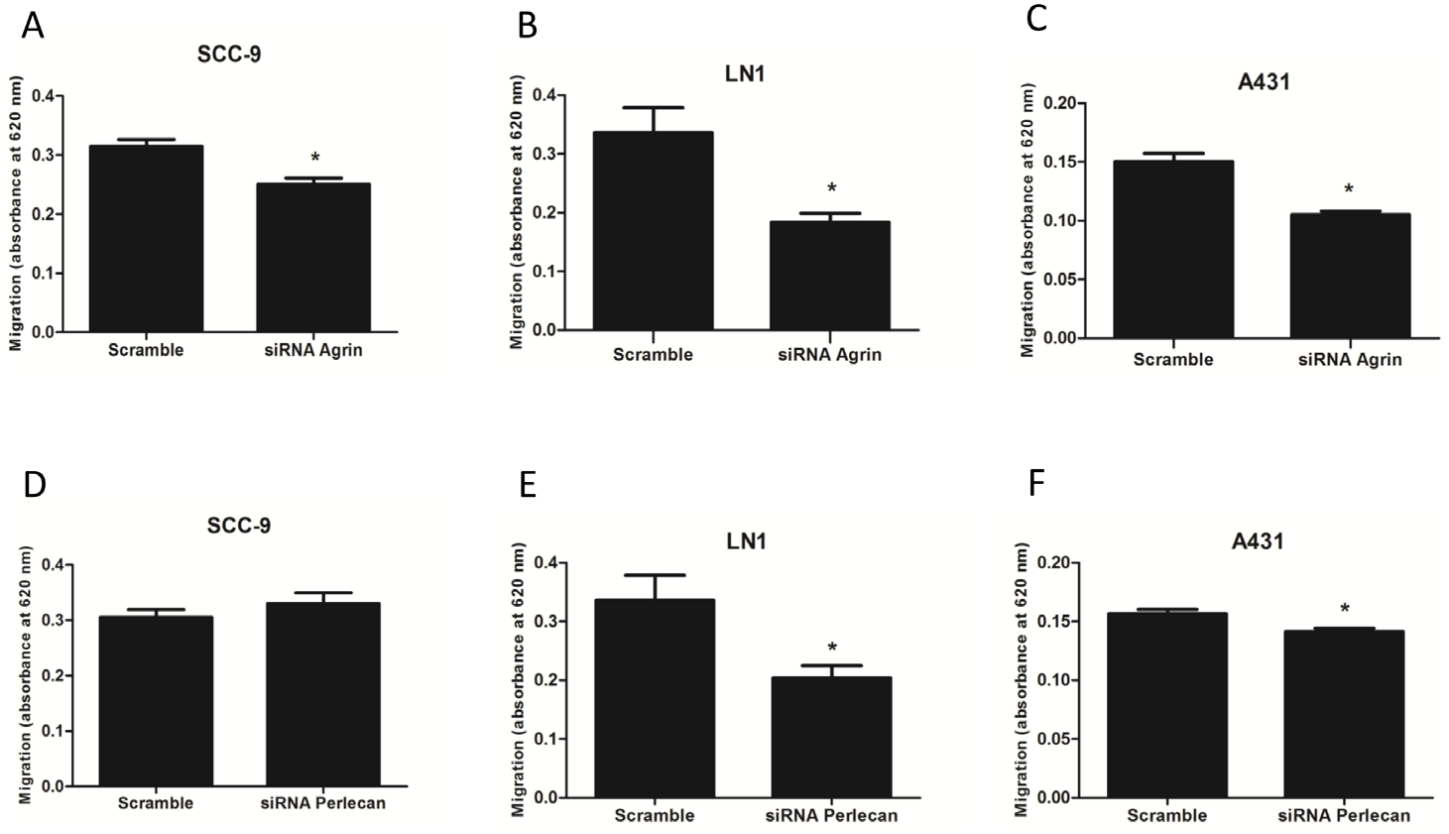
The role of agrin and perlecan in cell migration. Knockdown of agrin decreased migration of SCC-9 (n = 2, A), SCC-9 LN-1 (n = 3, B) and A431 (n = 3, C) cell lines. When perlecan was silenced by siRNA no difference was observed in SCC-9 migration (n = 2, D), but a significant reduction was observed in SCC-9 LN-1 (n = 3, E) and A431 (n = 3, F) migration (Student’s *t*-test, * indicates p<0.05).

The role of agrin and perlecan in cell migration was determined using transwell chamber. First, SCC-9, SCC-9 LN-1 and A431 cells treated with control siRNA (scramble) and siRNA against agrin or perlecan were plated in the upper chambers, and allowed to migrate towards the lower chamber containing medium supplemented with 1% FBS. For agrin silenced cells, migration was significantly diminished in SCC-9, SCC-9 LN-1 and A431 cells compared to scrambled control (Fig. 4A–D, n = 3, Student’s *t*-test, p<0.001). For perlecan silenced cells, migration was significantly diminished in SCC-9 LN-1 and A431 cells compared to scrambled control.

**Figure 5:**
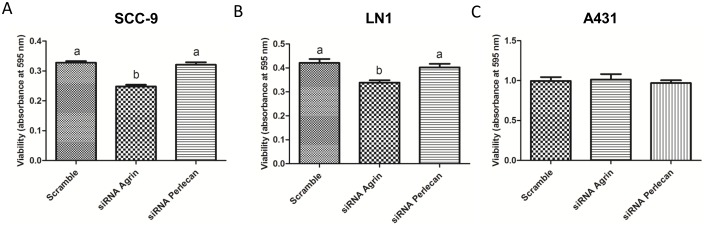
The role of agrin and perlecan in cell viability. The viability of SCC-9 (n = 2, A) and SCC-9 LN-1 (n = 3, B) was significantly reduced after siRNA-knockdown of agrin, but no difference in viability was observed in perlecan knockdown. The viability of A431 was not altered neither by agrin knockdown nor by perlecan knockdown (n = 3, C) (One-way ANOVA followed by Tukey’s test, different letters indicate statistically difference at p<0.05).

Cell viability was tested using MTT assay in the presence of 10% FBS, and we verified that SCC-9 and SCC-9 LN-1-agrin knockdown had a significant reduction in cell viability (Fig. 5A, n = 3, One-way ANOVA, followed by Tukey’s test, p<0.001). In the A431 knockdown cells no significant difference was observed for agrin- or perlecan-siRNA, compared to the scrambled-siRNA (Fig. 5B).

### The role of agrin and perlecan in cisplatin cell resistance

We sought to determine the cisplatin cell resistance in SCC-9, SCC-9 LN-1 and A431 when agrin or perlecan was silenced in these cell lines. It was observed that the siRNA-knockdown of agrin promoted a reduction in the cisplatin cell resistance in all cell lines used in this study: SCC-9 (2.4 fold), SCC-9 LN-1 (3.8 fold) and A431 (1.7 fold) (S3 Figure). On the other hand, when perlecan was silenced, no significant difference was observed in cisplatin cell resistance for SCC-9, SCC-9 LN-1 and A431 cell lines compared with scrambled (S3 Figure).

**Figure 6:**
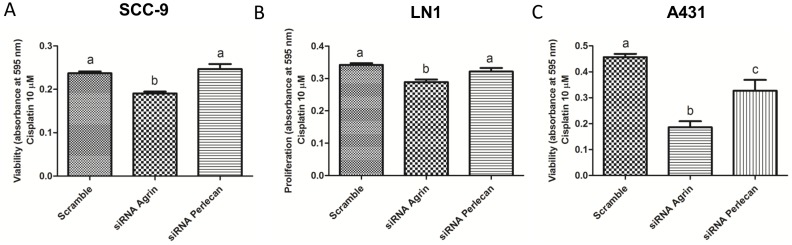
Viability of SCC-9 LN-1 and A431 cell lines in 10 µM of cisplatin treatment. At 10 µM of cisplatin, the viability of SCC-9 (n = 3, A), SCC-9 LN-1 (n = 3, B) and A431 (n = 3C) was significantly reduced when agrin was silenced. Only in A431 cell line the same result of decreasing in viability was observed for perlecan siRNA-knockdown (C) (One-way ANOVA followed by Tukey’s test, different letters indicate statistically difference at p<0.05).

At a concentration of 10 µM of cisplatin, SCC-9, SCC-9 LN-1 and A431 cell lines showed a significant reduction in cell viability when agrin expression was silenced (Fig. 6A and B, n = 3, ANOVA followed by Tukey’s test, p<0.05). A significant reduction in cell viability was also observed just in A431 perlecan-knockdown cells treated with 10 µM of cisplatin (Fig. 6C, n = 3, ANOVA followed by Tukey’s test, p<0.05).

## Discussion

Proteoglycans (PGs), essential macromolecules of the tumor microenvironment, have their expression altered during malignant transformation and tumor progression [9]. Agrin and perlecan are two of the major HSPG identified in the basement membrane, and their functional roles in modulation of cancer growth have been reviewed elsewhere [22]. In this study, we showed for the first time that agrin and perlecan are highly expressed in OSCCs, and the function of these proteins in oral cancer associated processes was investigated. Not only the expression of agrin and perlecan was shown to be higher in OSCC tissues compared to control tissues, but also their expression might be associated with different sites of origin, where higher expression of agrin was found in cell line originated from primary site (SCC-9), whereas higher expression of perlecan was found in cell line originated from metastatic site (SCC-9 LN-1). The spread of cancer cells from a primary tumor to form metastases at distant sites is a complex process that remains poorly defined [29], but it has been reported to involve detachment of cells from the tumor tissue, regulation of cell motility and invasion, proliferation and evasion through the lymphatic system or blood vessels [30]. Efforts have been made to elucidate tumor-related proteins that could influence the appearance of metastases in oral squamous cell carcinoma [31]–[33], which occur in about 40% of patients with oral cancer [34]. Therefore, the study of altered molecules in cell lines originated from distinct sites is essential to understand the molecular basis of this process.

GAGs polysaccharide chains are the main contributors to the proteoglycan functional properties and essential part of the matured proteoglycan molecules [35]. Besides, it was reported that glycans play a crucial role at various pathophysiological steps of cancer progression [36], especially by acting as co-receptors to stabilize growth-factor receptor signaling complexes and enhancing integrin-mediated cell adhesion, motility and intracellular signaling. The disruption of GAGs modification by heparanase was shown to facilitate tumor cell invasion [37] angiogenesis and metastasis [38]. In order to further understand the role of chondroitin sulfate modification in oral cancer, SCC-9 LN-1 cell lines were treated with chondroitinase and tested in adhesion and migration processes. Interestingly, when SCC-9 LN-1 cells were treated with chondroitinase, they had reduced ability to adhere to extracellular matrix proteins. It is important to mention that this event may be a result of the disruption of many cell surface chondroitin sulfate proteoglycans, such as syndecans, chondroitin sulfate proteoglycan 4, betaglycan, neuropilin-1, receptor protein tyrosine phosphatase, integrin and VEGFR-2, which were also previously demonstrated to be overexpressed in cancer [39].

It is well established that based upon their direct involvement in cell–cell and cell–ECM interactions, PGs have been strongly implicated in the regulation of cell movement [40]. However, how PGs actually affect this process is only partially understood and in some instances, controversial. In this study, we have demonstrated that agrin and perlecan play a role in the oral cancer cell movement by silencing agrin and perlecan, which promoted a strongly reduced in the ability of SCC-9 and SCC-9 LN-1 cell line to migrate and to adhere to matrigel.

Perlecan has been associated with the induction of cellular proliferation, differentiation and angiogenesis by interacting with a number of growth factors including FGFs 1, 2, 7, 9, and 18; hepatocyte growth factor, platelet derived growth factors-AA and -BB, and VEGF [14], [41]. Perlecan also exhibits adhesive [42] or anti-adhesive [43] properties presumably by differentially affecting surface receptors such as α2β1 integrin [44], [45]. Agrin is also able to interact with integrins [46], however there is still very limited acknowledgement on how agrin signals through the integrin receptors and how these interactions influence cell behavior.

Therapeutic PGs- and GAG-targeting modifications have been considered as anti-invasion and tumor-specific drug delivery potential approaches [47]. It was reported that both heparan sulfate and chondroitin sulfate modifications are able to interact with cisplatin and mediate entry-pathway [28], [48]. Platinum-based chemotherapy has been used for treating a wide variety of solid tumors, including lung, head and neck, ovarian, cervical, and testicular cancers for over three decades [49]. However, the emergence of drug resistance may limit the effectiveness of platinating agents in solid tumors [50]–[52], including OSCCs [53]. Recently, novel extracellular matrix cisplatin-resistant biomarkers from epithelial ovarian carcinoma (EOC) were identified using secretome analysis from EOC cell lines [54]. In our study, we showed new evidences of increasing cisplatin-sensibility by disrupting chondroitin sulfate modification, or reduction of agrin and perlecan proteins levels, suggesting it may have a potential target for therapeutic intervention.

In summary, we have identified a relevant role of agrin and perlecan in oral cancer cell adhesion, migration and cisplatin cell resistance, opening new perspectives for further investigations and targeting innovative and/or complementary therapeutic strategies.

## Supporting Information

S1 Figure **Immunohistochemistry analysis of agrin in tissue microarray.** Agrin showed higher expression in OSCC (n = 47) compared with normal mucosa (n = 10) (Mann-Whitney U test, p<0.0005). In lower panel, two representative figures show the higher expression of agrin in OSCC (B) compared with normal mucosa (A).
(TIF)

S2 Figure **Confirmation by qRT-PCR of silencing of agrin in SCC-9 (A), SCC-9 LN-1 (B) and A431 (C) and perlecan in SCC-9 (D), SCC-9 LN-1 (E) and A431 (F).** The data were normalized with the (glyceraldehyde-3-phosphate dehydrogenase gene was used as internal reference). Each bar represents mean ± SD of at least two independent experiments in triplicates.
(TIF)

S3 Figure **The role of agrin and perlecan in cisplatin cell resistance.** (A) SCC-9/control (scrambled, IC50 = 14.57 µM), SCC-9/siRNA Agrin (IC50 = 6.02 µM) and SCC-9/siRNA Perlecan (IC50 = 12.68 µM) were treated with increasing concentrations of cisplatin (0–100 µM) for 48 h and the IC50 concentrations were calculated using dose response curves generated by GraphPad Prism software. (B) SCC-9 LN-1/control (scrambled, IC50 = 17.65 µM), SCC-9 LN-1/siRNA Agrin (IC50 = 4.662 µM) and SCC-9 LN-1/siRNA Perlecan (IC50 = 15.55 µM) were treated with increasing concentrations of cisplatin (0–100 µM) for 48 h and the IC50 concentrations were calculated using dose response curves generated by GraphPad Prism software. (C) A431/control (scrambled, IC50 = 6.59 µM), A431/siRNA Agrin (IC50 = 3.85 µM) andA431/siRNA Perlecan (IC50 = 6.65 µM) were treated with increasing concentrations of cisplatin (0–100 µM) for 48 h and the IC50 concentrations were calculated using dose-response curves generated by GraphPad Prism software.
(TIF)

S1 Table Clinicopathological characteristics of the patients with OSCC in the TMA.
(XLSX)

S2 Table Clinicopathological characteristics of the patients with OSCC used in the qRT-PCR analysis.
(XLSX)
