# Correction: Agrin and Perlecan Mediate Tumorigenic Processes in Oral Squamous Cell Carcinoma

**DOI:** 10.1371/journal.pone.0119247

**Published:** 2015-03-18

**Authors:** The PLOS ONE Staff

The sixth author’s name is spelled incorrectly. The correct name is: César Rivera. The correct citation is: Kawahara R, Granato DC, Carnielli CM, Cervigne NK, Oliveria CE, Rivera C, et al. (2014) Agrin and Perlecan Mediate Tumorigenic Processes in Oral Squamous Cell Carcinoma. PLoS ONE 9(12): e115004. doi:10.1371/journal.pone.0115004
