# Supplementary figures and images for: Agrin and Perlecan Mediate Tumorigenic Processes in Oral Squamous Cell Carcinoma
Source: PLoS One. 2014 Dec 15;9(12):e115004. doi: 10.1371/journal.pone.0115004 (PMC4266612; doi:10.1371/journal.pone.0115004)

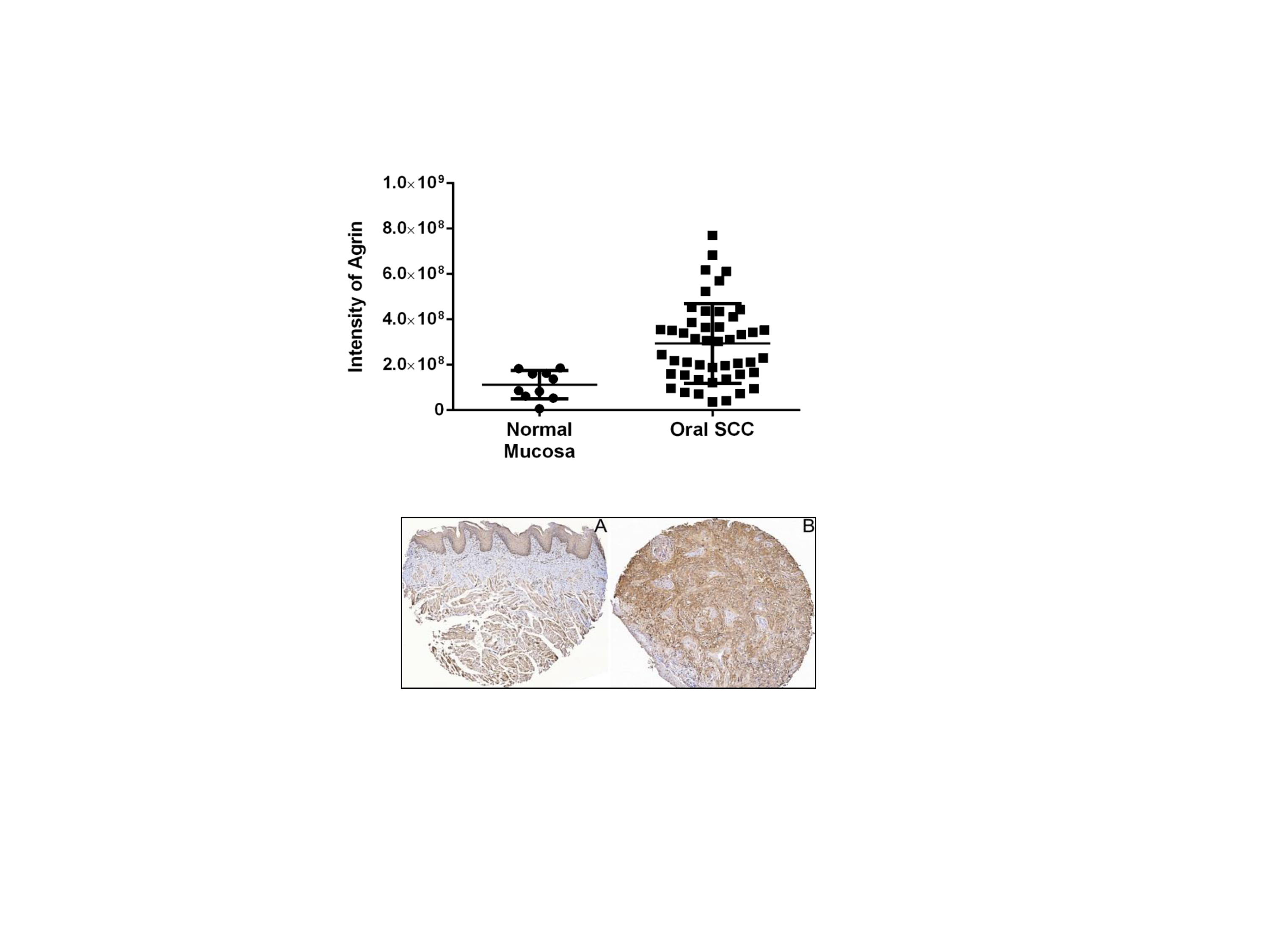

Supplement: S1 Figure — Immunohistochemistry analysis of agrin in tissue microarray. Agrin showed higher expression in OSCC (n = 47) compared with normal mucosa (n = 10) (Mann-Whitney U test, p<0.0005). In lower panel, two representative figures show the higher expression of agrin in OSCC (B) compared with normal mucosa (A). (TIF) [file pone.0115004.s001.tif]

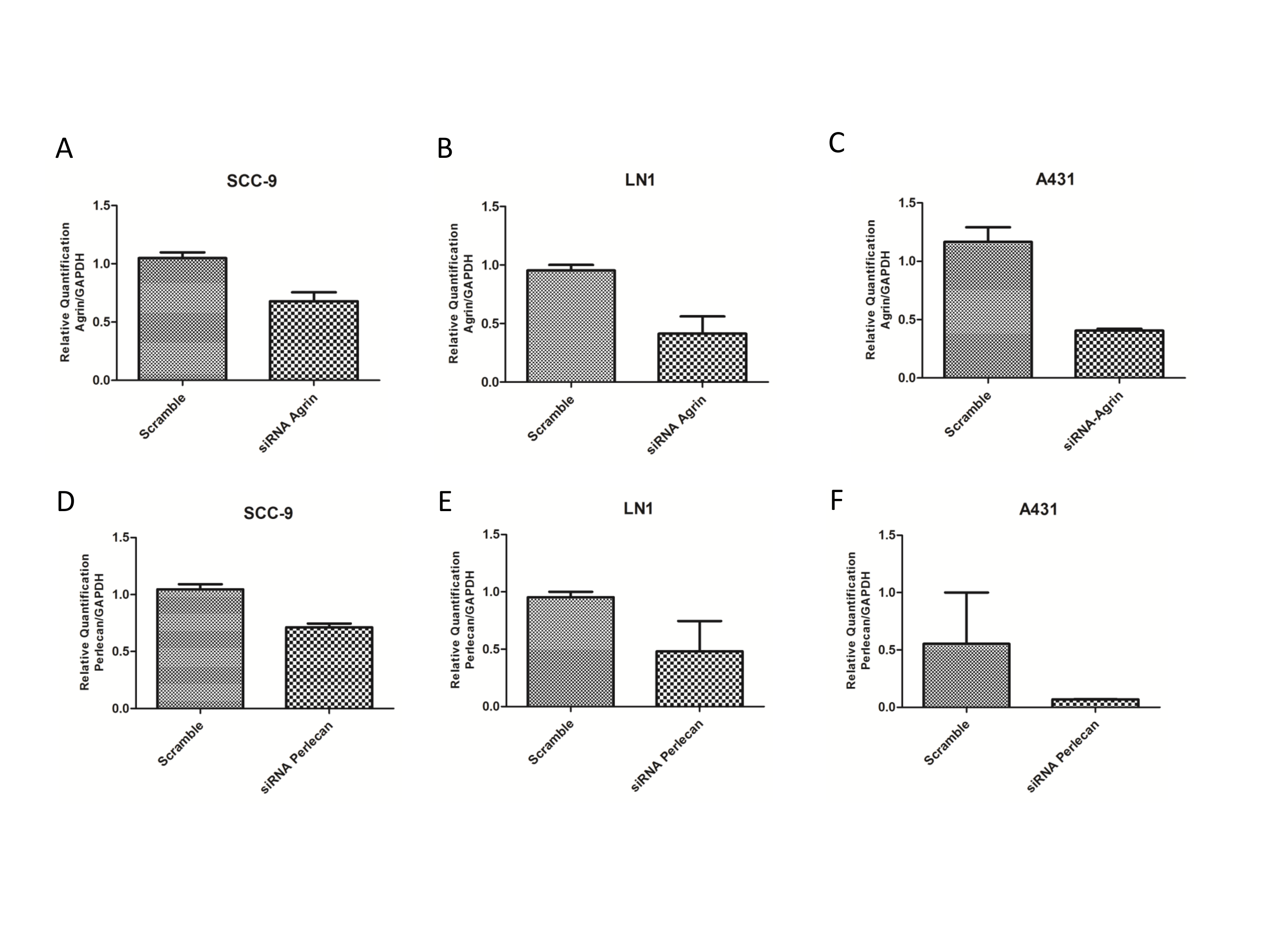

Supplement: S2 Figure — Confirmation by qRT-PCR of silencing of agrin in SCC-9 (A), SCC-9 LN-1 (B) and A431 (C) and perlecan in SCC-9 (D), SCC-9 LN-1 (E) and A431 (F). The data were normalized with the (glyceraldehyde-3-phosphate dehydrogenase gene was used as internal reference). Each bar represents mean ± SD of at least two independent experiments in triplicates. (TIF) [file pone.0115004.s002.tif]

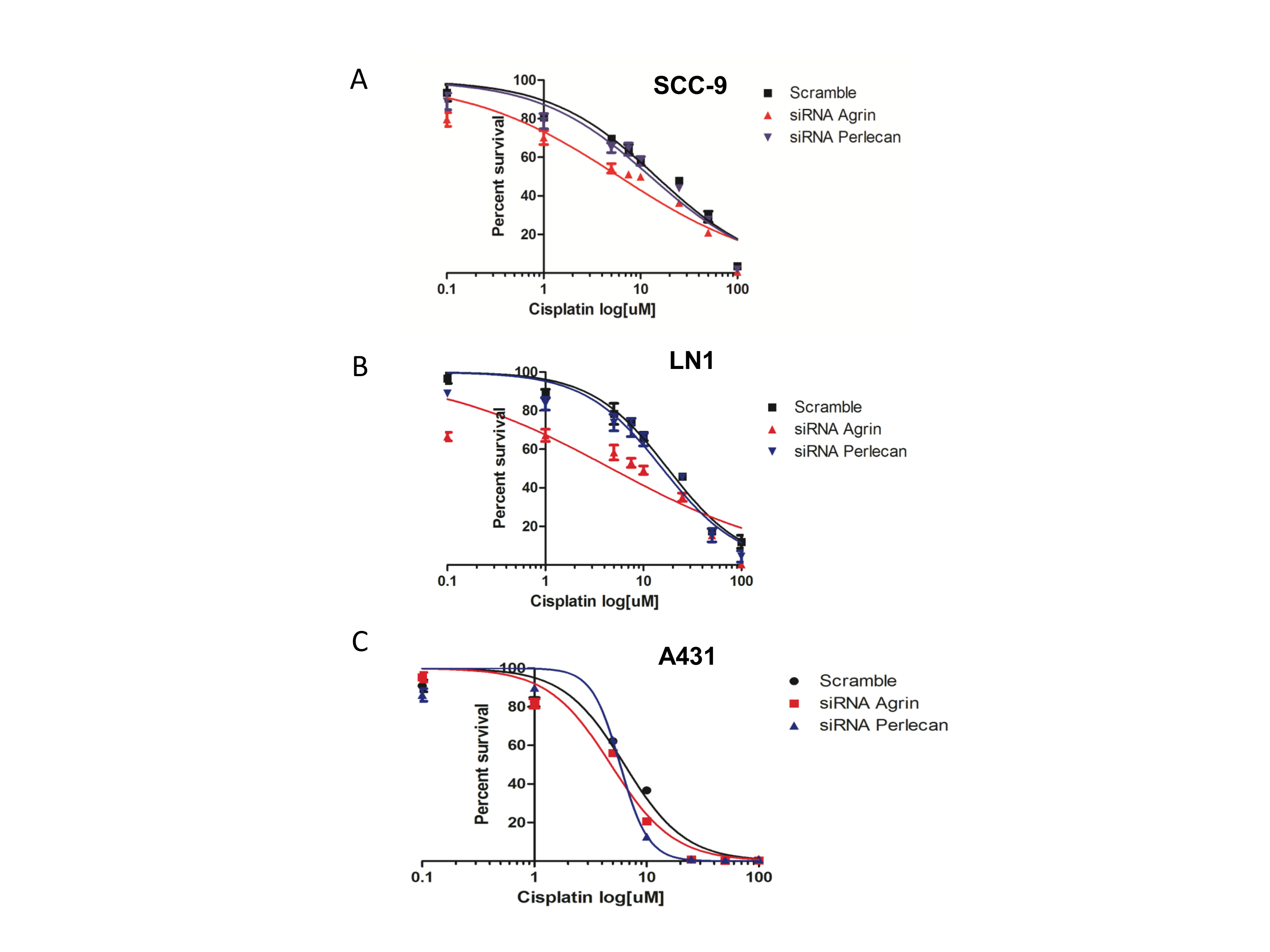

Supplement: S3 Figure — The role of agrin and perlecan in cisplatin cell resistance. (A) SCC-9/control (scrambled, IC50 = 14.57 µM), SCC-9/siRNA Agrin (IC50 = 6.02 µM) and SCC-9/siRNA Perlecan (IC50 = 12.68 µM) were treated with increasing concentrations of cisplatin (0–100 µM) for 48 h and the IC50 concentrations were calculated using dose response curves generated by GraphPad Prism software. (B) SCC-9 LN-1/control (scrambled, IC50 = 17.65 µM), SCC-9 LN-1/siRNA Agrin (IC50 = 4.662 µM) and SCC-9 LN-1/siRNA Perlecan (IC50 = 15.55 µM) were treated with increasing concentrations of cisplatin (0–100 µM) for 48 h and the IC50 concentrations were calculated using dose response curves generated by GraphPad Prism software. (C) A431/control (scrambled, IC50 = 6.59 µM), A431/siRNA Agrin (IC50 = 3.85 µM) andA431/siRNA Perlecan (IC50 = 6.65 µM) were treated with increasing concentrations of cisplatin (0–100 µM) for 48 h and the IC50 concentrations were calculated using dose-response curves generated by GraphPad Prism software. (TIF) [file pone.0115004.s003.tif]
